# Global transcriptome profiling reveals root- and leaf-specific responses of barley (*Hordeum vulgare* L.) to H_2_O_2_

**DOI:** 10.3389/fpls.2023.1223778

**Published:** 2023-09-12

**Authors:** Sabarna Bhattacharyya, Maya Giridhar, Bastian Meier, Edgar Peiter, Ute C. Vothknecht, Fatima Chigri

**Affiliations:** ^1^ Institute for Cellular and Molecular Botany, University of Bonn, Bonn, Germany; ^2^ Leibniz Institute for Food Systems Biology at the Technical University of Munich, Freising, Germany; ^3^ Institute of Agricultural and Nutritional Sciences, Faculty of Natural Sciences III, Martin Luther University Halle-Wittenberg, Halle, Germany

**Keywords:** barley, H_2_O_2_, oxidative stress, RNA-sequencing, reactive oxygen species (ROS), transcriptome profiling, stress response

## Abstract

In cereal crops, such as barley (*Hordeum vulgare* L.), the ability to appropriately respond to environmental cues is an important factor for yield stability and thus for agricultural production. Reactive oxygen species (ROS), such as hydrogen peroxide (H_2_O_2_), are key components of signal transduction cascades involved in plant adaptation to changing environmental conditions. H_2_O_2_-mediated stress responses include the modulation of expression of stress-responsive genes required to cope with different abiotic and biotic stresses. Despite its importance, knowledge of the effects of H_2_O_2_ on the barley transcriptome is still scarce. In this study, we identified global transcriptomic changes induced after application of 10 mM H_2_O_2_ to five-day-old barley plants. In total, 1883 and 1001 differentially expressed genes (DEGs) were identified in roots and leaves, respectively. Most of these DEGs were organ-specific, with only 209 DEGs commonly regulated and 37 counter-regulated between both plant parts. A GO term analysis further confirmed that different processes were affected in roots and leaves. It revealed that DEGs in leaves mostly comprised genes associated with hormone signaling, response to H_2_O_2_ and abiotic stresses. This includes many transcriptions factors and small heat shock proteins. DEGs in roots mostly comprised genes linked to crucial aspects of H_2_O_2_ catabolism and oxidant detoxification, glutathione metabolism, as well as cell wall modulation. These categories include many peroxidases and glutathione transferases. As with leaves, the H_2_O_2_ response category in roots contains small heat shock proteins, however, mostly different members of this family were affected and they were all regulated in the opposite direction in the two plant parts. Validation of the expression of the selected commonly regulated DEGs by qRT-PCR was consistent with the RNA-seq data. The data obtained in this study provide an insight into the molecular mechanisms of oxidative stress responses in barley, which might also play a role upon other stresses that induce oxidative bursts.

## 1 Introduction

In aerobic organisms, reactive oxygen species (ROS) are generated as by-products of certain metabolic pathways in plant organelles such as chloroplasts, mitochondria, and peroxisomes (Huang et al., 2019; Smirnoff and Arnaud, 2019). Because of their high reactivity with cellular components, aerobic organisms have developed systems for enzymatic ROS removal based on the activity of ascorbate peroxidase (APX), superoxide dismutase (SOD), and catalase (CAT) as well as non-enzymatic antioxidative systems such as ascorbic acid, proline, and glutathione (GSH) (Foyer and Noctor, 2003; Ahmad et al., 2010). Plants also actively produce ROS as part of signaling cascades that coordinate the appropriate responses to environmental stimuli and contribute to stress tolerance (Pei et al., 2000; Zhu, 2016; Mohanta et al., 2018). It is proposed that systemic communication *via* redox systems is very fundamental to all photosynthetic organisms.

The ROS species hydrogen peroxide (H_2_O_2_) has been shown to play a role in various processes such as cell differentiation, senescence, and cell wall formation (Kärkönen and Kuchitsu, 2015; Ribeiro et al., 2017; Zeng et al., 2017). It is generated from superoxide in various cellular compartments as well as the apoplast as a result of a highly conserved superoxide dismutation reaction (Smirnoff and Arnaud, 2019). H_2_O_2_ is also known to be transported across the cell membrane by specific aquaporins (Bienert et al., 2007) and to participate in long distance cell signaling (Mittler et al., 2011). Exogenous treatment with H_2_O_2_ has been shown to increase the tolerance of plants to abiotic stress by regulating multiple stress-responsive pathways and expression of genes including heat shock proteins and genes involved in abscisic acid (ABA) biosynthesis (Wahid et al., 2007; Terzi et al., 2014). An activation of ROS-dependent signaling by H_2_O_2_ causes the accumulation of defense proteins such as ROS-scavenging enzymes, transcription factors (TFs), and other response factors (Hossain et al., 2015), and it thus increases the tolerance of plants to abiotic stress. For example, certain HEAT SHOCK TRANSCRIPTION FACTORS (HSFs) have been suggested to serve as sensors that perceive H_2_O_2_ and regulate the expression of oxidative stress response genes (Miller and Mittler, 2006).

An early transcriptomic approach pursued to elucidate the effect of H_2_O_2_ was performed in *Arabidopsis thaliana* cell suspension cultures and showed that various TFs, hormone-associated pathways, and genes associated with other vital metabolic pathways like photosynthesis and fatty acid biosynthesis were affected (Desikan et al., 2001). Other studies revealed the role of H_2_O_2_ as a signaling molecule in a variety of plant species and under various conditions. For instance, H_2_O_2_ is involved in the response of plants to a variety of environmental cues, such as salt stress in tomato (Li et al., 2019), heat stress in rice (Wang et al., 2014), chilling stress in mung beans and manila grass (Yu et al., 2003; Wang et al., 2010), copper stress in maize and mung bean (Guzel and Terzi, 2013; Fariduddin et al., 2014), and many more (Khan et al., 2018).

Barley is one of the oldest cultivated cereal crops and has a high tolerance to stresses like salt, drought, and heat (Munns et al., 2006; Rollins et al., 2013; Gürel et al., 2016). Whereas changes in the barley transcriptome upon those stresses have been analyzed (Janiak et al., 2018; Osthoff et al., 2019; Nefissi Ouertani et al., 2021), a global transcriptome analysis in response to H_2_O_2_ has not been performed yet.

In the present study, we used RNA sequencing (RNA-Seq) to analyze changes in the transcriptome of barley roots and leaves upon application of H_2_O_2_. This analysis identified a total of 1001 and 1883 differentially expressed genes (DEGs) in response to H_2_O_2_ in leaves and roots, respectively. Comparative and quantitative analyses of gene expression patterns revealed commonly regulated key genes related to H_2_O_2_ stress between both tissues, nine of which were further confirmed by qRT-PCR analysis. The data obtained in this study contribute to the understanding of molecular mechanisms of oxidative stress response in barley, which might also play a role upon other stresses that induce oxidative bursts.

## 2 Materials and methods

### 2.1 Plant material and growth conditions

Barley plants *(Hordeum vulgare* cultivar Golden Promise) were grown in pots filled with water-soaked vermiculite in a climate-controlled growth chamber under long-day conditions with 16 h light at 20°C and a light intensity of 120 µmol photons m^-2^ s^-1^ (Philips TLD 18W of alternating 830/840 light color temperature) and 8 h darkness at 18°C for five days.

### 2.2 H_2_O_2_ application and RNA isolation

Five-day-old seedlings were harvested and washed carefully to remove any remaining vermiculite prior to submersion in 10 mM H_2_O_2_ (Carl Roth, Germany) or ddH_2_O (control) for three hours. The duration of H_2_O_2_ treatment was selected based on previous studies, which showed that at this time point H_2_O_2_ induced the strongest changes in the expression of most of the H_2_O_2_-responsive genes (Desikan et al., 2001; Stanley Kim et al., 2005; Hieno et al., 2019). Subsequently, seedlings were carefully rinsed with ddH_2_O and dissected into roots and leaves. Samples were shock-frozen in liquid nitrogen and homogenized using a sterile, ice-cold mortar and pestle. Total RNA was extracted using the Quick-RNA miniprep Kit (Zymo Research, USA) according to the manufacturer’s instructions. The yield and purity of extracted RNA was determined with a NABI Nanodrop UV/Vis Spectrophotometer (MicroDigital, South Korea). The integrity of the extracted RNA was verified by separation of the 28S and 18S rRNA bands on a 1% agarose gel.

### 2.3 RNA-sequencing and data analyses

RNA sequencing was performed on three biological replicates for each treatment. Each replicate furthermore consisted of pooled material from three plants. Library preparation and transcriptome sequencing (3’ mRNA sequencing) were carried out at the NGS Core Facility (Medical Faculty at the University of Bonn, Germany) using a NOVASEQ 6000 (Illumina, USA) with a read length of 1x100 bases and an average sequencing depth of >10 million raw reads per sample (**Table 1**). 3’ end sequencing libraries were prepared using the QuantSeq protocol (Moll et al., 2014). Briefly, oligo dT priming were followed by synthesis of the complementary first strand without any prior removal of ribosomal RNA. After successful introduction of Illumina specific adapter sequences, the resulting cDNA was further purified with magnetic beads. The unpaired reads were processed for quality control using fastQC and cutAdapt (Martin, 2011) in order to trim any remaining adapter sequences. They were then aligned using Tophat2 software (Trapnell et al., 2012) against a *H. vulgare* IBSC v2 reference genome obtained from Ensembl (http://plants.ensembl.org/info/data/ftp/index.html) using a Bowtie index (Langmead and Salzberg, 2012) created with the help of the reference genome (in FASTA format; the individual FASTA files of the chromosomes were concatenated using the “cat” command in UNIX shell). The alignment with Tophat2 was performed on an Ubuntu 18.04 LTS operating system, in a UNIX shell environment. Every step after alignment was performed using R 4.0.0 (R Core Team, 2020). Gene counts from the aligned BAM files were generated using featureCounts function in RStudio (Liao et al., 2014). Differential gene expression analyses was carried out using DESeq2 (Love et al., 2014). The p-values were corrected using the False Discovery Rate (FDR) method (Benjamini and Hochberg, 1995) and subsequently the FDR and the log_2_FC cutoffs were set to 0.01 and 1, respectively. Principal Component Analyses (PCA) plots were prepared with the raw gene counts for all samples and replicates using the tidyverse and ggplot2 packages. The volcano plots and heatmaps were generated using the EnhancedVolcano and Pheatmap packages, respectively. In addition, transcript per million (TPM) values of each gene were calculated using a separate function designed in the R environment (**Supplementary Table S1**). With common regulated DEGs, a clustering was performed with four predefined clusters based on FDR and log_2_FC cutoffs of 0.01 and 0.5, respectively. The first and second cluster consisted of commonly down- and up-regulated genes, respectively, while the third and fourth cluster contained counter-regulated genes between leaves and roots of barley. The clusters were then represented as heatmaps using the pheatmap package and line plots using the ggpubr package.

**Table 1:** Summary of total reads and aligned reads in the RNA-seq samples from barley roots and leaves obtained under H_2_O_2_ treatment and control conditions.

Sample	Replicate	Total Reads	Aligned Reads	% Aligned Reads
**root control**	RC1	15222810	12333400	81.02
	RC2	13555021	10223311	75.42
	RC3	12544002	9988003	79.62
**leaf control**	LC1	12392862	9242908	74.58
	LC2	14067426	10125991	71.98
	LC3	12314839	9224084	74.90
**root + H_2_O_2_**	RT1	12123370	8559783	70.61
	RT2	13079745	9303393	71.13
	RT3	12698432	10154310	79.97
**leaf + H_2_O_2_**	LT1	13222658	11555866	87.39
	LT2	14555200	12333012	84.73
	LT3	12220331	10214419	83.59

For each treatment three biological replicates were performed, each containing the combined RNA from three plants. LC-Leaf control, LT-Leaf H_2_O_2_ treated, RC-Root control, and RT-Root H_2_O_2_ treated.

Gene ontology (GO) and enrichment analyses were carried out using shinyGO (Ge et al., 2020). Categories were chosen as significant if the FDR was less than 0.05 (Benjamini and Hochberg, 1995). Homology searches against the *A. thaliana* genome were carried out using the BaRT (Barley Reference Transcript) tool available on www.ics.hutton.ac.uk (Mascher et al., 2017) based on a E-value cutoff of 1e^-30^.

### 2.4 Quantification of transcript levels by qRT-PCR

qRT-PCR was performed with three replicates for each sample. Each replicate consisted of the pooled RNA material from three different plants. Synthesis of first strand cDNA for qRT-PCR was carried out from at least 1 µg of total RNA using the RevertAid first strand cDNA synthesis kit (Thermo Fisher Scientific, USA) with oligo-dT_18_ primers following the manufacturer’s instructions. The quality of cDNA was assessed using a NABI UV/Vis Nanodrop Spectrophotometer. Gene expression was quantified in 48-well plates using a BioRad CFX 96 real-time PCR detection system (BioRad, Germany) and a SYBR Green PCR master mix (Thermo Fisher Scientific, USA). All forward and reverse primers used for qRT-PCR are listed in **Supplementary Table S2**. Data were quantified using the BioRad CFX Maestro software, and the expression was estimated using the 2^–δδCt^ method (Livak and Schmittgen, 2001) after normalization against the two reference genes *HvACTIN* and *HvGAPDH*, as the C_q_ values of both genes were unchanged upon H_2_O_2_ treatment. Data were analyzed statistically with one-way analysis of variance (ANOVA) and Tukey’ Post-Hoc HSD test using the agricolae and tidyverse packages, respectively. Graphs were prepared using the ggpubr package.

### 2.5 H_2_O_2_ staining and microscopic analyses

Staining of hydrogen peroxide in barley leaves and roots was performed with 2’,7’-dichlorodihydrofluorescein diacetate (H_2_-DCFDA; Thermo Fisher Scientific, USA) based on a modified protocol (Kaur et al., 2016). Briefly, five-day-old barley seedlings were treated with either 10 mM H_2_O_2_ or ddH_2_O (control) for 3 hours. Afterwards, the seedlings were briefly rinsed and treated with 10 µM H_2_-DCFDA prepared from a 4 mM stock dissolved in DMSO for 1 hour in the dark. After staining, seedlings were washed, and roots and leaves were mounted separately on a microscopy slide. 2’,7’-Dichlorfluorescein (DCF) fluorescence was analyzed using a Leica SP8 Lightning confocal laser scanning microscope (Leica Microsystems, Germany). For excitation, an argon laser with a wavelength of 488 nm was used, and emission of 517-527 nm was detected using a HyD Detector. Fluorescence intensity was quantified in regions of interest (ROI) using the integrated LASX software.

## 3 Results

### 3.1 Differential gene expression in leaves and roots of barley in response to application of H_2_O_2_

**Figure 1:**
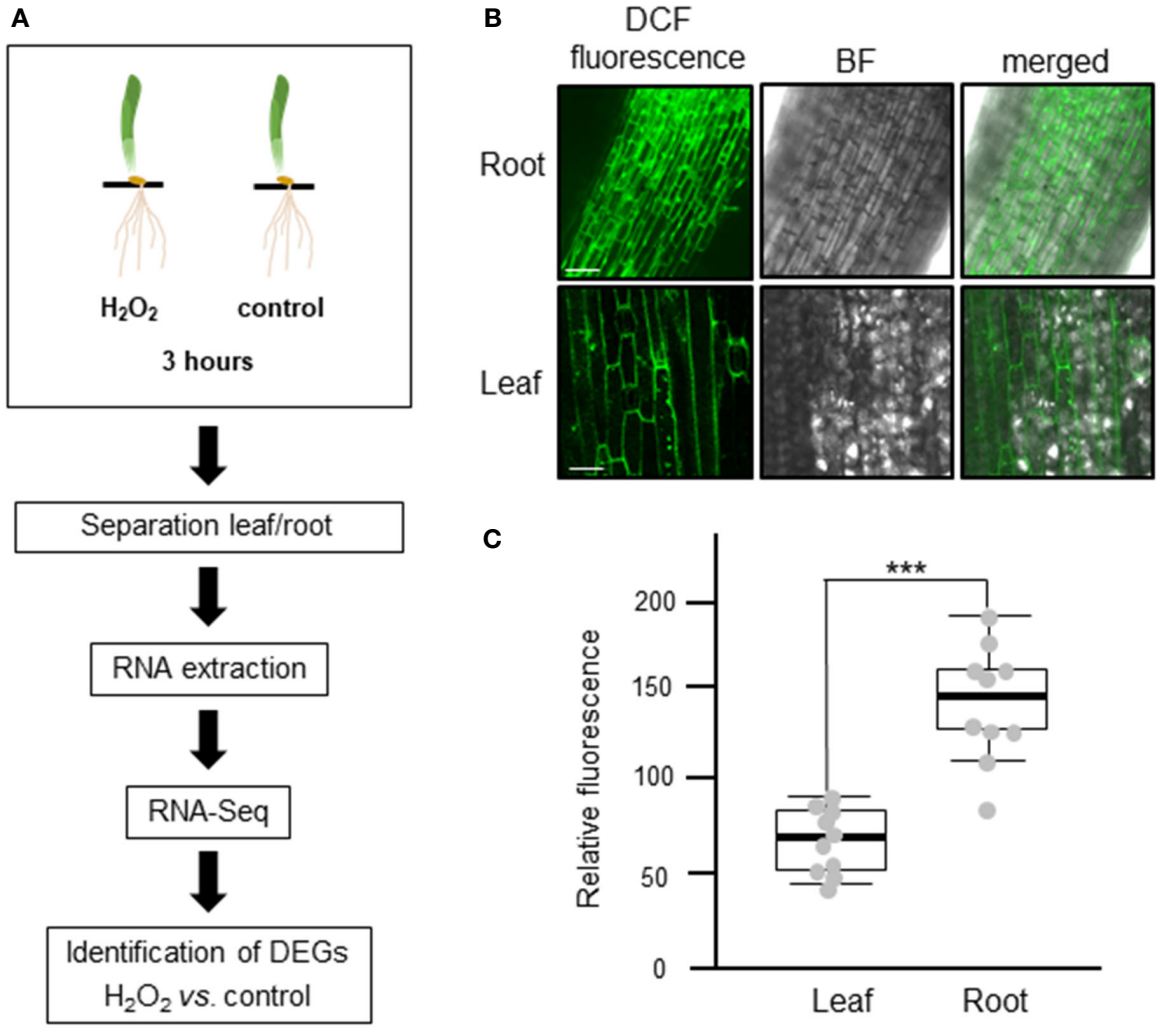
Experimental design to analyze the transcriptional changes of barley plants to oxidative stress. **(A)** Schematic representation of the study design. Five-day-old barley plants were treated with either 10 mM H_2_O_2_ or water (control) for three hours. After the treatment, leaves and roots were separated, RNA was extracted, and three independent biological replicates, each containing the pooled RNA from three plants, were submitted to RNA-Seq analyses. The raw reads obtained were subjected to quality control and aligned against the barley reference genome. Based on raw gene counts, a differential expression analysis was carried out using DESeq2. **(B)** Uptake of H_2_O_2_ in roots (upper panel) and leaves (lower panel) visualized by H_2_-DCFDA. Green fluorescence of the 2’,7’-Dichlorfluorescein (DCF) was observed using a Leica SP8 lightning confocal laser scanning microscope. BF: bright field; bar: 100 µm. **(C)** Quantification of fluorescence intensity of H_2_-DCFDA relative to untreated control tissues. Each dot represents the average of five regions of interests (ROIs). ROIs were taken from two independent images from three biological replicates (n=6). Statistical analysis was carried out using the two-tailed t-test (*** = P<0.001).

**Figure 2:**
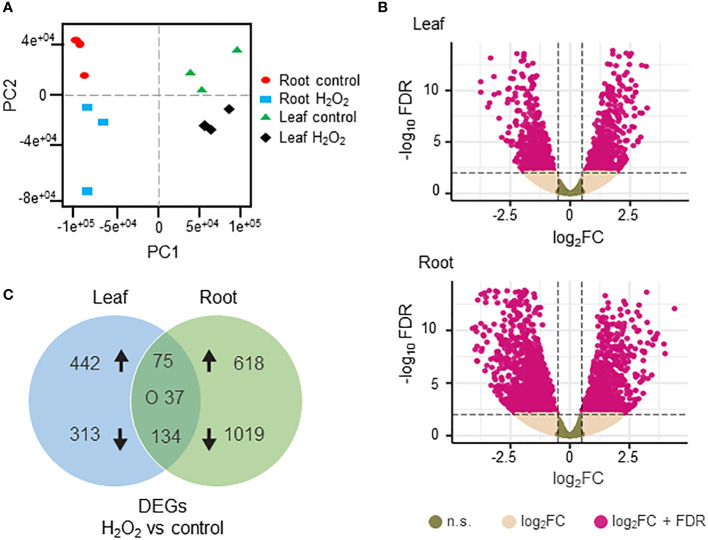
Differentially expressed genes (DEGs) in H_2_O_2_-treated and untreated barley plants. **(A)** Principal component analysis of the RNAseq data showing the homogeneity of the different samples. PC1 (X axis) separates the samples by tissue while PC2 (Y axis) separates the samples by treatment. **(B)** Volcano plots of the DEGs in leaves (upper panel) and roots (lower panel). The X axis represents the fold change (Log_2_FC) of the DEGs (H_2_O_2_ *vs.* control), whereas the Y axis represents the statistical significance (log_10_FDR). Pink dots indicate genes that fit the DESeq criteria of FDRand │Log_2_FCin│, while beige dots represent DEGs that fit only Log_2_FC. N.S.: not significant **(C)** Venn diagram representing DEGs (DESeq, adjusted to FDR<0.01 and │Log_2_FC│≥1) between H_2_O_2_-treated and untreated samples in leaves and roots. Arrows indicate up- and down-regulation. ‘O’ indicates counter-regulated genes.

To investigate the transcriptomic modulation in barley (*Hordeum vulgare* cv. Golden Promise) in response to oxidative stress, five-day-old plants were exposed for three hours to 10 mM H_2_O_2_ or to ddH_2_O as control (**Figure 1A**). H_2_-DCFDA staining confirmed that H_2_O_2_ penetrated both roots and leaves (**Figures 1B, C** and **Supplementary Figure 1**). RNA was then extracted separately from roots and leaves, and RNA-seq analysis was carried out on three biological replicates per tissue and treatment, each comprising the pooled RNA from three different plants (**Supplementary Table S1**). On average approximately 13 million total reads were obtained per sample. About 75-85% of these reads could be aligned to the barley reference genome (**Table 1**). To assess the main variances within the dataset, a principal component analysis (PCA) was performed. The result showed that PC1 (X-axis), which separates the samples by tissue, represents the largest variation in our dataset compared to PC2 (Y-axis), which separates the samples by treatment (**Figure 2A**). Consequently, the differential gene expression analysis was separately performed for the leaf and root samples.

Differentially expressed genes (DEGs) between H_2_O_2_-treated and control samples were identified based on fold change (FC) │Log_2_FC ≥ 1│ and FDR < 0.01 (**Supplementary Table S3**). A total number of 2884 DEGs were detected across both tissues. H_2_O_2_ application clearly resulted in stronger transcriptional changes in roots compared to leaves (**Figure 2B**). Of the 1883 DEGs detected in roots, 701 were up- and 1182 were down-regulated, while in leaves 1001 DEGs were identified with 546 up- and 455 down-regulated (**Figure 2C**). Among all DEGs only 75 and 134 were commonly up- and down-regulated, respectively, in both tissues, while 37 were counter-regulated.

### 3.2 Gene ontology analyses

**Figure 3:**
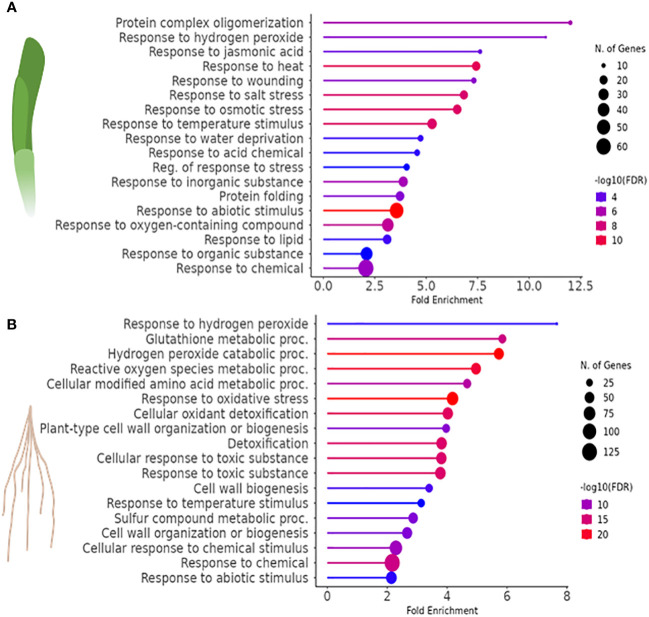
Gene ontology (GO) enrichment analysis to identify biological processes associated with the DEGs (FDR<0.01) of H_2_O_2_-treated *vs.* control samples in **(A)** leaves and **(B)** roots of barley.

GO classification was used to identify the 20 most significant biological process categories within the DEGs. The results show that not only the number of genes, but also the biological processes affected by H_2_O_2_ were clearly different between leaves and roots (**Figure 3**). In leaves, GO terms associated with genes that showed the highest fold change were related to protein complex oligomerization, response to H_2_O_2_ and jasmonate. Further categories with lower fold change but often higher number of genes comprised quite global stress effects associated with different, mostly abiotic stimuli, but also wounding (**Figure 3A**). In roots, many of the enriched GOs were associated with response to oxygenic stress including H_2_O_2_ catabolism, glutathione and ROS metabolism, or cellular oxidant detoxification as well as with cell wall modulation (**Figure 3B**).

#### 3.2.1 Differentially expressed genes in barley leaves in response to H_2_O_2_

In barley leaves, the most highly enriched GO term category upon exposure to H_2_O_2_ was the response to H_2_O_2_ and protein complex oligomerization (**Figure 3A**). Both categories consist of the same SMALL HEAT SHOCK PROTEINS (SHSP domain-containing proteins) (**Table 2**). SHSPs are ubiquitous in prokaryotic and eukaryotic organisms and function as chaperone proteins involved in the response to many abiotic stresses (Basha et al., 2012; Waters, 2013). Their expression levels were shown in different plant species to increase upon stress and to enhance stress tolerance. Here, barley leaves exposed to H_2_O_2_ showed an increased expression of SHSPs, except for the 18.8 kDa class V heat shock protein (HORVU2Hr1G046370), which was down-regulated. All of the differentially regulated SHSPs have close orthologs in Arabidopsis (Li and Liu, 2019) with the majority being orthologous to *AtHSP17.6II* (At5g12020).

**Table 2:** Selected DEGs associated with top GO terms in leaves of barley in response to H_2_O_2_.

*Category*	*Gene ID*	*log_2_FC*	*Functional protein*	*Predicted ortholog in* A. thaliana
**Response to H_2_O_2_/ protein complex oligomerization**				
	HORVU2Hr1G046370	-3.74	SHSP domain-containing protein	AT4G21870 (AtHSP15.4)
	HORVU3Hr1G020500	2.24	SHSP domain-containing protein	AT5G12020/AT5g12030 (AtHSP17.6)
	HORVU3Hr1G020490	3.03	SHSP domain-containing protein	AT5G12020/AT5g12030 (AtHSP17.6)
	HORVU3Hr1G020390	1.267	SHSP domain-containing protein	AT5G12020/AT5g12030 (AtHSP17.6)
	HORVU0Hr1G020420	1.54	SHSP domain-containing protein	AT5G37670 (AtHSP15.7)
	HORVU3Hr1G020520	1.84	SHSP domain-containing protein	AT5G12020/AT5g12030 (AtHSP17.6)
	HORVU6Hr1G082360	2.98	SHSP domain-containing protein	At1G54050 (AtHSP17.4)
**Response to jasmonic acid**				
	HORVU5Hr1G062290	2.34	TIFY domain-containing protein	AT1G74950 (AtJAZ12)
	HORVU4Hr1G076850	1.80	TIFY domain-containing protein	no homolog
	HORVU5Hr1G098090	1.21	Uncharacterized protein	AT1G13280 (AtAOC4)
	HORVU7Hr1G118010	-1.44	Oxidored FMN domain-containing	AT1G76680 (AtOPR1)
	HORVU2Hr1G004230	-1.55	Oxidored FMN domain- containing	AT1G76690 (AtOPR2)
	HORVU6Hr1G081000	0.76	Allene oxide synthase	AT5G42650 (AtCYP74A/AtAOS)
**Response to abiotic stimulus/ osmotic stress/ hormones**				
**Auxin**	HORVU7Hr1G084940	1.81	Auxin responsive protein	AT4G14550 (AtIAA14/AtSLR)
	HORVU5Hr1G087880	1.48	Auxin responsive protein	AT5G65980 (AtPILS7)
	HORVU7Hr1G033820	1.22	Auxin responsive protein	AT1G19220 (AtARF19)
	HORVU1Hr1G086070	1.00	Auxin responsive protein	no homolog
	HORVU1Hr1G086070	1.00	Auxin responsive protein	no homolog
	HORVU6Hr1G058890	-1.52	Auxin response factor	AT4G30080 (AtARF16)
	HORVU7Hr1G077110	-1.62	Auxin responsive protein	no homolog
	HORVU5Hr1G093580	-2.40	Auxin responsive protein	AT3G04730 (AtIAA16)
**Abcisic acid**	HORVU7Hr1G085130	2.34	Multiple protein bridging factor	AT3G24500 (AtMBF1c)
	HORVU7Hr1G035500	1.58	bZIP domain-containing protein	AT4G34000 (AtABF3/AtbZIP37)
	HORVU3Hr1G069590	1.37	HSF_domain-containing protein	AT3G24520 (AtHsfC1)
	HORVU6Hr1G028790	1.30	WRKY domain-containing protein	AT4G31800 (AtWRKY18)
	HORVU5Hr1G115100	1.03	GRAM domain-containing protein	At5G13200 (AtGEML5/AtGER5/AtGRE5)
**other**	HORVU5Hr1G097560	1.62	HTH MYB domain-containing protein	AT2G38090
	HORVU3Hr1G085180	1.26	MYB domain-containing protein	no homolog
	HORVU6Hr1G091700	-1.13	Ethylene receptor domain-containing protein	AT3G04580 (AtEIN4)
	HORVU4Hr1G077310	-1.31	AP2/ERF domain-containing protein	no homolog
	HORVU4Hr1G000700	-1.92	AP2/ERF domain-containing protein	AT3G23240 (AtERF092/AtERF1b)
	HORVU3Hr1G010190	-3.31	AP2/ERF domain-containing protein	AT1G68840 (AtEDF2/AtRAV2/AtTEM2)
**Photosynthesis**				
	HORVU6Hr1G091660	-1.67	Chlorophyll a-b binding protein	AT2G34420 (AtLHCb1.5)
	HORVU1Hr1G088920	-1.37	Chlorophyll a-b binding protein	AT2G34420 (AtLHCb1.5)
	HORVU7Hr1G040370	-1.16	Chlorophyll a-b binding protein	AT2G34420 (AtLHCb1.5)
	HORVU6Hr1G047870	-1.11	Ribulose bisphosphate carboxylase LSU	ATCG00490 (RubisCo LSU)
	HORVU5Hr1G109250	-1.07	Chlorophyll a-b binding protein	AT1G29930 (AtLHCb1.3)
	HORVU5Hr1G109260	-0.93	Chlorophyll a-b binding protein	AT2G34420 (AtLHCb1.5)
	HORVU2Hr1G040780	-0.92	Chlorophyll a-b binding protein	AT5G54270 (AtLHCb3)
	HORVU1Hr1G078380	-0.91	Chlorophyll a-b binding protein	AT2G34420 (AtLHCb1.5)
	HORVU2Hr1G060880	-0.87	PsbP domain-containing protein	AT1G06680 (AtPsP1)
	HORVU5Hr1G100140	-0.81	PSI-F	AT1G31330 (AtPsaF)
	HORVU7Hr1G046320	-0.72	Chlorophyll a-b binding protein	AT3G54890 (AtLHCa1)
	HORVU3Hr1G009210	-0.71	PSI subunit V	AT4G12800 (AtPsaL)
	HORVU1Hr1G088870	-0.68	Chlorophyll a-b binding protein	AT2G34430 (AtLHCb1.4)

An enrichment was also found for genes involved in hormone biosynthesis and signaling, especially jasmonate, auxin, and abscisic acid (ABA). Jasmonate-related DEGs were represented by the specific GO-term category ‘response to jasmonic acid’. This category comprised two up-regulated TIFY domain-containing proteins with no direct homologs in Arabidopsis (**Table 2**). The TIFY domain is also known as ZIM domain which is present in members of the transcriptional repressor JASMONATE ZIM-domain (JAZ) family, key elements in the jasmonate signaling pathway (Chung and Howe, 2009; Pauwels and Goossens, 2011). This category also includes genes that encode for enzymes involved in jasmonate biosynthesis (Schaller and Stintzi, 2009; Bittner et al., 2022) such as ALLENE OXIDE CYCLASE (*AOC*), and OXOPHYTODIENOATE-REDUCTASE (*OPR*) as well as ALLENE OXIDE SYNTHASE (*AOS*) but with a FC less than 2 (FC 1.69, Log_2_FC=0.76). By contrast, genes related to other hormone signaling pathways were found redundantly interspersed in the two GO terms ‘response to abiotic stimulus’ and ‘response to salt stress’ (**Figure 3A**). With regard to auxin, a number of orthologs to auxin-responsive genes from Arabidopsis, especially IAA-type TFs, were found. Similar to the jasmonate signaling pathway, H_2_O_2_ seems to affect the auxin pathway differentially since both, up- and down-regulated DEGs, were identified. All components related to the phytohormone ABA were up-regulated and those related to APETALA2/ETHYLENE RESPONSIVE FACTOR (*AP2/ERF*) domain-containing proteins, known to be involved in abiotic stress responses and associated with various hormones, were down-regulated. Similar to the GO term categories related to auxin, both sets comprise mostly orthologs to TFs or co-regulators known in Arabidopsis (**Table 2**).

In leaves, genes associated with photosynthesis light harvesting in photosystem I, were also affected, however, the category did not appear in the top GOs since for several of the genes the FC was less than 2 but mostly higher than 1.5 (**Table 2**; Log_2_FC between 0.5 and 1). This category contained mostly down-regulated DEGs, including several orthologs of Arabidopsis LHCII trimer components, i.e., genes encoding for LHCb1 and LHCb3, and the LHCa1 protein. It furthermore comprised orthologs to the photosystem I subunits PSAF and PSAL but also the oxygen evolving complex subunit PSBP-1 and the large subunit of RIBULOSE-1,4-BISPHOSPHATE-CARBOXYLASE/OXYGENASE (Rubisco) (**Table 2**).

#### 3.2.2 Differentially expressed genes in barley roots in response to H_2_O_2_

In barley roots, the most enriched GO terms are associated with response to oxidative stress and detoxification (**Figure 3B**). This is also evident by the fact that many DEGs within those GO terms are class-III peroxidases, catalases, or genes related to glutathione metabolism, which were grouped together as a category named ‘Detoxification of H_2_O_2_’ (**Table 3**). In plants, class-III peroxidases have been described in association with a wide variety of biotic and abiotic stresses along with plant defense mechanisms (Almagro et al., 2009; Shigeto and Tsutsumi, 2016). While most peroxidases were up-regulated, some were down-regulated along with a number of glutathione transferases, an ascorbate peroxidase (*APX*), and *CATALASE 1*. We also found strong up-regulation of the genes for two putative detoxification efflux carriers/multidrug and toxic compound extrusion (*DTX/MATE*) transporters. These metabolite transporters have been described to be associated with plant stress responses and overexpression of a gene encoding a cotton DXT protein in Arabidopsis reduced stress-induced levels of H_2_O_2_ (Lu et al., 2019).

**Table 3:** Selected DEGs associated with top GO terms in roots of barley in response to H_2_O_2_.

*Category*	*Gene ID*	*log_2_FC*	*Functional annotation*	*Predicted ortholog in* A. thaliana
**Response to H_2_O_2_**				
	HORVU0Hr1G020420	-1.21	SHSP domain containing protein	AT5G37670 (AtHSP15.7)
	HORVU2Hr1G077710	-1.59	SHSP domain containing protein	AT4G10250 (AtHSP22)
	HORVU3Hr1G006940	-2.24	SHSP domain containing protein	No ortholog
	HORVU3Hr1G020390	-1.92	SHSP domain containing protein	AT5G12020 (AtHSP17.6II)
	HORVU3Hr1G020490	-2.79	SHSP domain containing protein	AT5G12020 (AtHSP17.6II)
	HORVU3Hr1G020520	-2.96	SHSP domain containing protein	AT5G12020 (AtHSP17.6II)
	HORVU4Hr1G015170	-3.2	SHSP domain containing protein	AT4G10250 (AtHSP22)
	HORVU4Hr1G060720	-1.34	SHSP domain containing protein	AT3G46230 (AtHSP17.4)
	HORVU4Hr1G060760	-2.88	SHSP domain containing protein	AT1G53540 (AtHSP17.6C)
	HORVU6Hr1G008640	-2.55	Catalase	AT1G20630 (AtCAT1)
	HORVU7Hr1G014870	-1.54	ABC transporter domain containing protein	AT1G31770 (AtABCG14)
**Detoxification of H_2_O_2_**				
**H_2_O_2_ catabolism**	HORVU7Hr1G039550	3.97	Peroxidase	AT1G05260 (AtPRX3)
	HORVU2Hr1G026640	3.65	Peroxidase	AT1G05260 (AtPRX3)
	HORVU7Hr1G010280	3.598	Peroxidase	AT4G11290 (AtPRX39)
	HORVU1Hr1G016730	2.96	Peroxidase	AT2G18140 (AtPRX14)
	HORVU2Hr1G018550	2.91	Peroxidase	AT5G05340 (AtPRX52)
	HORVU7Hr1G039590	2.74	Peroxidase	AT1G05260 (AtPRX3)
	HORVU2Hr1G018530	2.60	Peroxidase	AT5G05340 (AtPRX52)
	HORVU7Hr1G039570	2.21	Peroxidase	AT1G05260 (AtPRX3)
	HORVU0Hr1G002840	2.17	Peroxidase	AT4G11290 (AtPRX39)
	HORVU2Hr1G100610	2.07	Peroxidase	AT5G17820 (AtPRX57/AtPRXR10)
	HORVU1Hr1G016770	2.01	Peroxidase	AT4G11290 (AtPRX39)
	HORVU2Hr1G026590	1.93	Peroxidase	AT4G11290 (AtPRX39)
	HORVU2Hr1G026520	1.84	Peroxidase	AT4G11290 (AtPRX39)
	HORVU2Hr1G026540	1.83	Peroxidase	AT4G11290 (AtPRX39)
	HORVU6Hr1G026600	1.67	Peroxidase	AT5G05340 (AtPRX52)
	HORVU7Hr1G039560	1.52	Peroxidase	AT1G05260 (AtPRX3)
	HORVU1Hr1G016870	-1.84	Peroxidase	AT5G66390 (AtPRX72/AtPRXR8)
	HORVU2Hr1G124930	-1.99	Peroxidase	AT1G71695 (AtPRX12/AtPRXR6)
	HORVU4Hr1G022280	-2.15	Peroxidase	AT5G05340 (AtPRX52)
**Glutathione metabolism**	HORVU6Hr1G063830	-1.47	Glutathione peroxidase	AT4G11600 (AtGPX6/AtGPXL6)
	HORVU5Hr1G006330	-1.17	Glutathione transferase	no homolog
	HORVU1Hr1G049230	-1.28	Glutathione transferase	AT2G29470 (AtGSTU3)
	HORVU1Hr1G021140	-1.36	Glutathione transferase	AT3G62760 (AtGSTF13)
	HORVU6Hr1G011120	-2.16	GST_C terminal domain-containing protein	AT4G19880
	HORVU5Hr1G006330	-1.17	Glutathione transferase	no homolog
	HORVU1Hr1G049070	-2.86	GST_N terminal domain-containing protein	AT1G10370 (AtGSTU17)
**Response to ROS / Detoxification**	HORVU4Hr1G057170	-1.31	APX domain-containing protein	AT1G07890 (AtAPX1/AtC3H)
	HORVU6Hr1G008640	-2.55	Catalase	AT1G20630 (AtCAT1)
	HORVU4Hr1G011690	2.26	DTX/MATE metabolite transporter	AT3G26590 (AtDTX29)
	HORVU0Hr1G022350	-4.09	DTX/MATE metabolite transporter	AT5G52450 (AtDTX16)
**Cell wall**				
	HORVU4Hr1G028720	2.70	Xyloglucan endotransglucosylase/ hydrolase	AT5G13870 (AtXTH5/AtXTR12)
	HORVU2Hr1G010800	2.37	ExpansinA11	AT1G20190 (AtEXPA11)
	HORVU3Hr1G116470	2.07	Pectin acetylesterase	no homolog
	HORVU3Hr1G016820	2.04	Xyloglucan endotransglucosylase/ hydrolase	AT5G57550 (AtXTH25)
	HORVU2Hr1G120100	1.47	Endoglucanase	AT1G48930 (AtGH9C1/AtCEL6)
	HORVU3Hr1G016800	1.44	Xyloglucan endotransglucosylase/ hydrolase	AT5G57550 (AtXTH25)
	HORVU5Hr1G118270	1.43	Cellulose synthase	AT5G64740 (AtCESA6/AtIRX2)
	HORVU7Hr1G093680	1.27	Expansin	AT4G38210 (AtEXPA20)
	HORVU7Hr1G098370	1.55	Xyloglucan endotransglycosylase	AT4G25810 (AtXTH23/AtXTR6)
	HORVU3Hr1G091360	257	Pectin esterase	AT5G09760 (AtPME51)

As in leaves, the most highly enriched GO term category in roots upon exposure to H_2_O_2_ was the response to H_2_O_2_, albeit with very few genes (**Figure 3B**). Similar to leaves, this category includes several SHSP domain-containing proteins, but in contrast to leaves, they were down-regulated (**Table 3**). All of the differentially regulated SHSPs have close orthologs in Arabidopsis, with several of them being orthologous to AtHSP17.6. This category contains also down-regulated catalase and ABC transporter containing domain proteins.

H_2_O_2_ treatment also induced up-regulation of components of cell wall biogenesis and modulation, such as xyloglucan endotransglucosylase/hydrolase, expansin, endo-1,4-beta glucanase, pectin acetyl esterase, and cellulose synthase (**Table 3**) that were found interspersed in several GO term categories. Indeed, H_2_O_2_ and peroxidases were shown to be involved in cell wall remodeling upon environmental stress (Tenhaken, 2015).

### 3.3 Common DEGs of leaves and roots in response to H_2_O_2_

**Figure 4:**
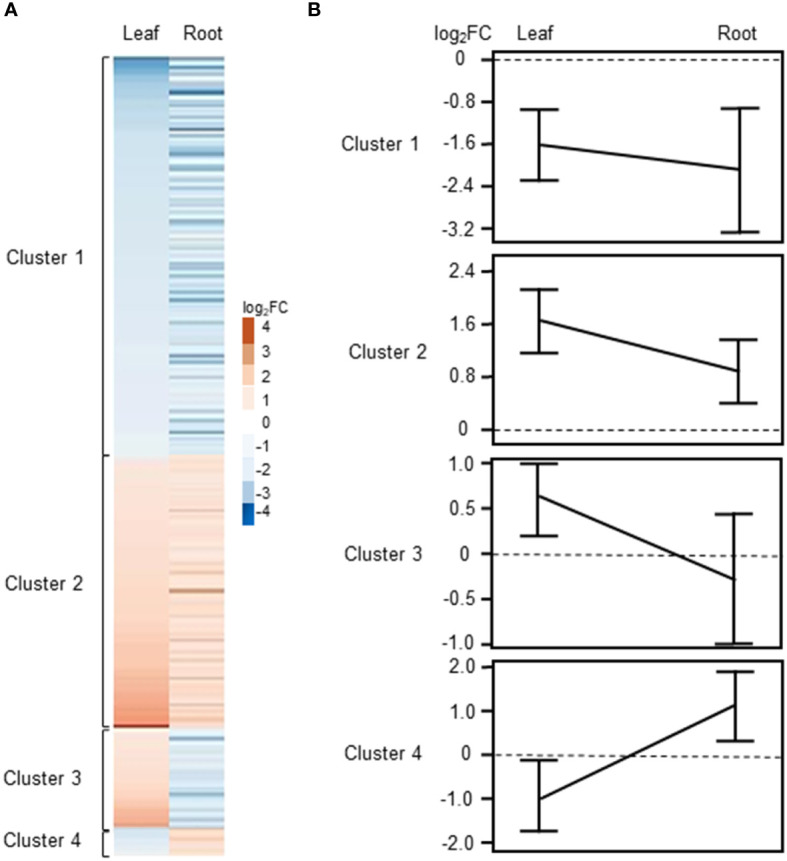
Clustering of DEGs commonly regulated or counter-regulated in leaves and roots of barley upon H_2_O_2_ treatment (|Log_2_FC|≥0.5 and FDR<0.01). **(A)** Heat map showing the Log_2_FC associated with each gene in leaves and roots. **(B)** Line plot showing the mean ± SE of the |Log_2_FC| associated with each cluster in leaves and roots.

As described above, we identified a total of 246 common DEGs between leaves and roots of barley when using a │log_2_FC ≥ 1│cutoff (**Supplementary Table S3**, **Figure 2C**). For several genes, we noticed that they were differentially regulated in both tissues, however, in one tissue they showed an expression with a FC>2 (│log_2_FC ≥ 1│) while in the other tissue a FC less than 2 but higher as 1.5 Thus, for (│log_2_FC between 1 and 0.5│) was detected. determination of commonly regulated genes in leaves and roots we used a cutoff of Log_2_FC≥0.5 and listed these genes separately in **Supplementary Table S3**. Using this cut-off, a total 349 common DEGs were identified between roots and leaves of barley (**Supplementary Figure S2**; **Supplementary Table S3**). Of these, 116 and 176 genes were up- and down-regulated, respectively, while 58 genes showed counter-regulation. These common DEGs were organized in four clearly distinguishable clusters (**Figure 4A**), with either commonly down- (cluster 1) and up-regulated (cluster 2) genes or genes up-regulated in leaves but down-regulated in roots (cluster 3) and *vice versa* (cluster 4). Heat maps and line plots were constructed to visualize the changes in gene expression pattern for each cluster (**Figures 4A, B**).

#### 3.3.1 Commonly up- and down-regulated genes

**Figure 5:**
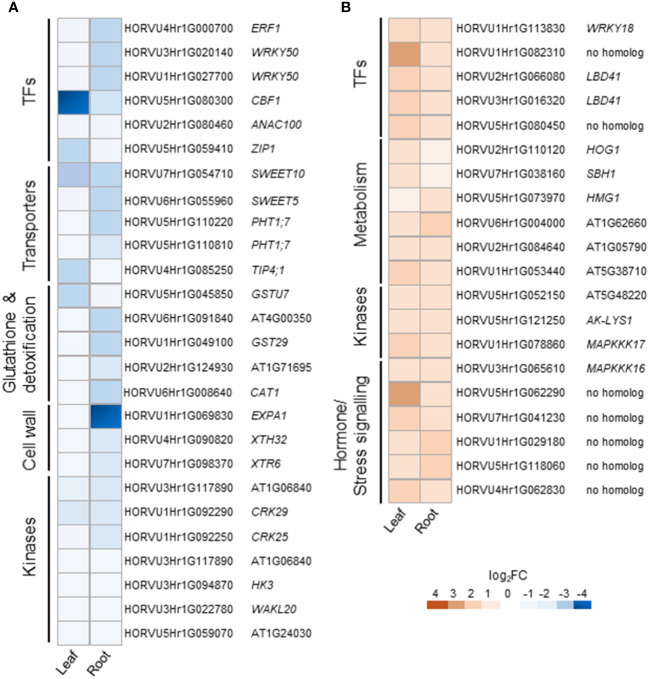
Selected DEGs commonly regulated in leaves and roots of barley upon H_2_O_2_ treatment. Down-regulated **(A)** and up-regulated **(B)** genes are grouped by functional category and presented with their Arabidopsis orthologs. TFs, transcription factors.

Cluster 1 contains DEGs commonly down-regulated in leaves and roots upon H_2_O_2_ treatment (**Supplementary Table S3**), among them members of important transcription factors such as *AP2/ERF*, *WRKY*, *CBF1*, *NAC*, and *HD-ZIP HOMEOBOX* (**Supplementary Table S4**, **Figure 5A**). Cluster 1 also comprises orthologs to the Arabidopsis sugar transporters *SWEET10* and *SWEET5*. Other transporters were orthologs to the phosphate transporter *PHT1;7* and the aquaporin *TIP4;1*. TIP aquaporins in plants had been shown to not only transport water molecules but also other molecules like H_2_O_2_ (Kurowska et al., 2020). In addition to components of oxidative stress, detoxification or cell wall biogenesis and modification that were already discussed in chapter 3.2.2, cluster 1 also contained several kinases including orthologs to the *CYSTEINE-RICH RECEPTOR-LIKE PROTEIN KINASES* (CRKs), *CRK29* and *CRK25*. *CRKs* are presented in Arabidopsis by a large gene family with over 40 members and have been associated with various abiotic and biotic stresses (Bourdais et al., 2015).

Cluster 2 contains DEGs commonly up-regulated in leaves and roots (**Supplementary Table S3**). Interestingly, it contains TFs of similar families as cluster 1, like *WRKY* and *AP2/ERF* but also orthologs of the LOB DOMAIN CONTAINING PROTEIN 41 (*LBD41*) from Arabidopsis (**Supplementary Table S4**; **Figure 5B**). DEGs associated with primary metabolism like amino acid and nucleic acid metabolism were also found in cluster 2. Genes associated with primary metabolism were also shown to be up-regulated in other transcriptome studies associated with abiotic stress (Hirai et al., 2004; Wang et al., 2014) and DEGs found in cluster 2 do not seem to be related to any specific metabolic pathway. Two MITOGEN-ACTIVATED PROTEIN KINASEs (MAPKs) identified in cluster 2 are orthologs to *AtMAPKKK16* and *AtMAPKKK17*, both of which were shown to be regulated by ABA (Wang et al., 2011).

#### 3.3.2 Counter-regulated genes

**Figure 6:**
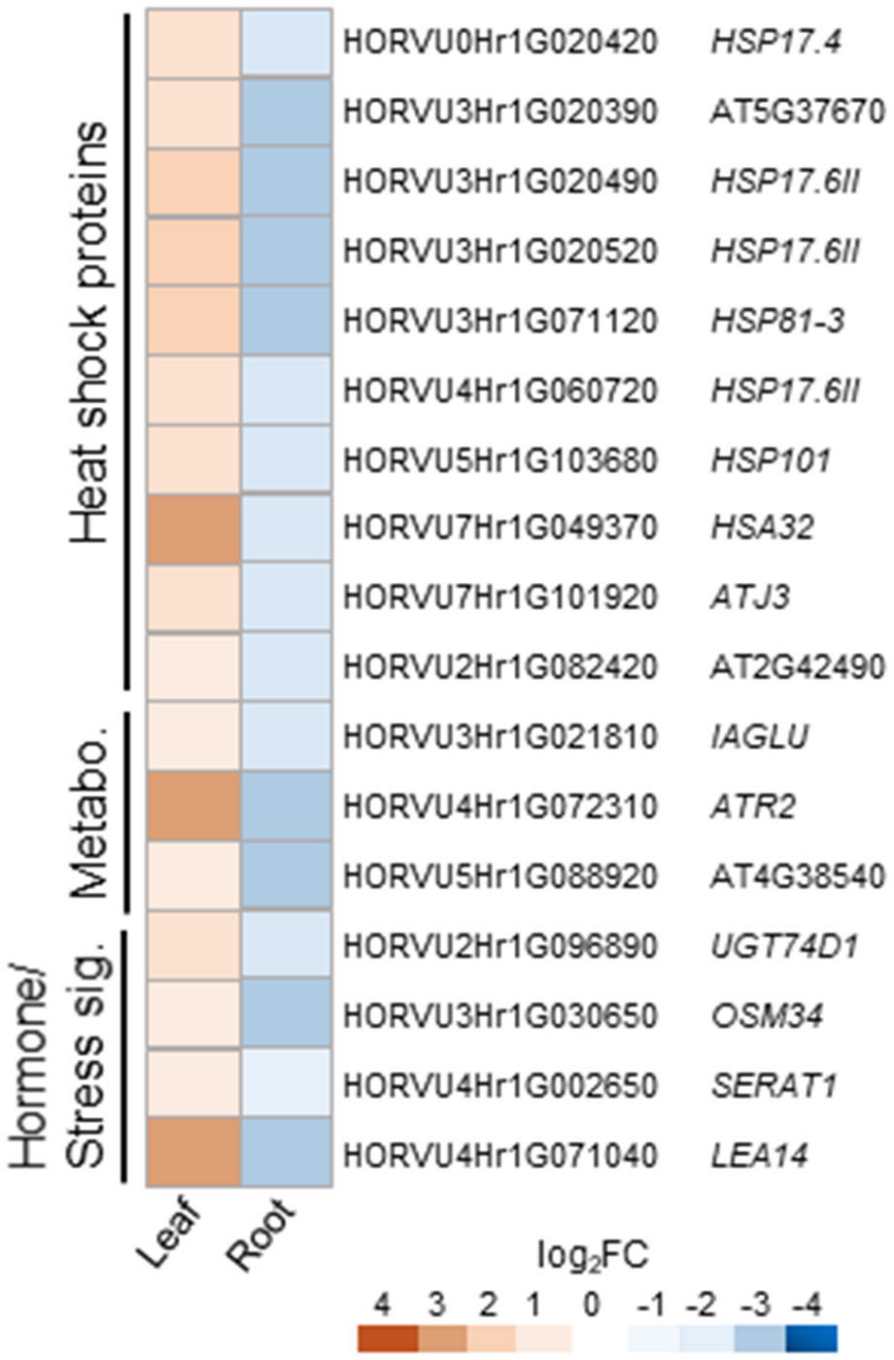
Selected counter-regulated DEGs in leaves and roots upon H_2_O_2_ treatment. Genes up-regulated in leaves and down-regulated in roots are grouped by functional category and presented with their Arabidopsis orthologs. Metabo., metabolism; sig., signaling.

Cluster 3 consists of 42 DEGs up-regulated in leaves and down-regulated in roots of barley upon H_2_O_2_ treatment (**Supplementary Table S3**). Nine of these DEGs are orthologs to different small heat shock proteins from Arabidopsis (**Supplementary Table S4**; **Figure 6**). The cluster furthermore comprises an assorted set of genes whose orthologs in Arabidopsis are connected with various metabolic pathways and hormone signaling.

Cluster 4 consists of only 15 genes and no common functional categories were found (**Supplementary Table S4**). However, they include genes, whose Arabidopsis orthologs have been associated with hormones, or cell wall modification, i.e. the *COPPER-CONTAINING AMINE OXIDASE 3 (CUAO3)* that was suggested to be involved in stress response since it was up-regulated upon treatment with several hormones or flagellin (Planas-Portell et al., 2013).

Overall, clusters 3 and 4 show very few genes previously described to be associated with oxidative stress.

### 3.4 qRT-PCR confirmation of selected DEGs

**Figure 7:**
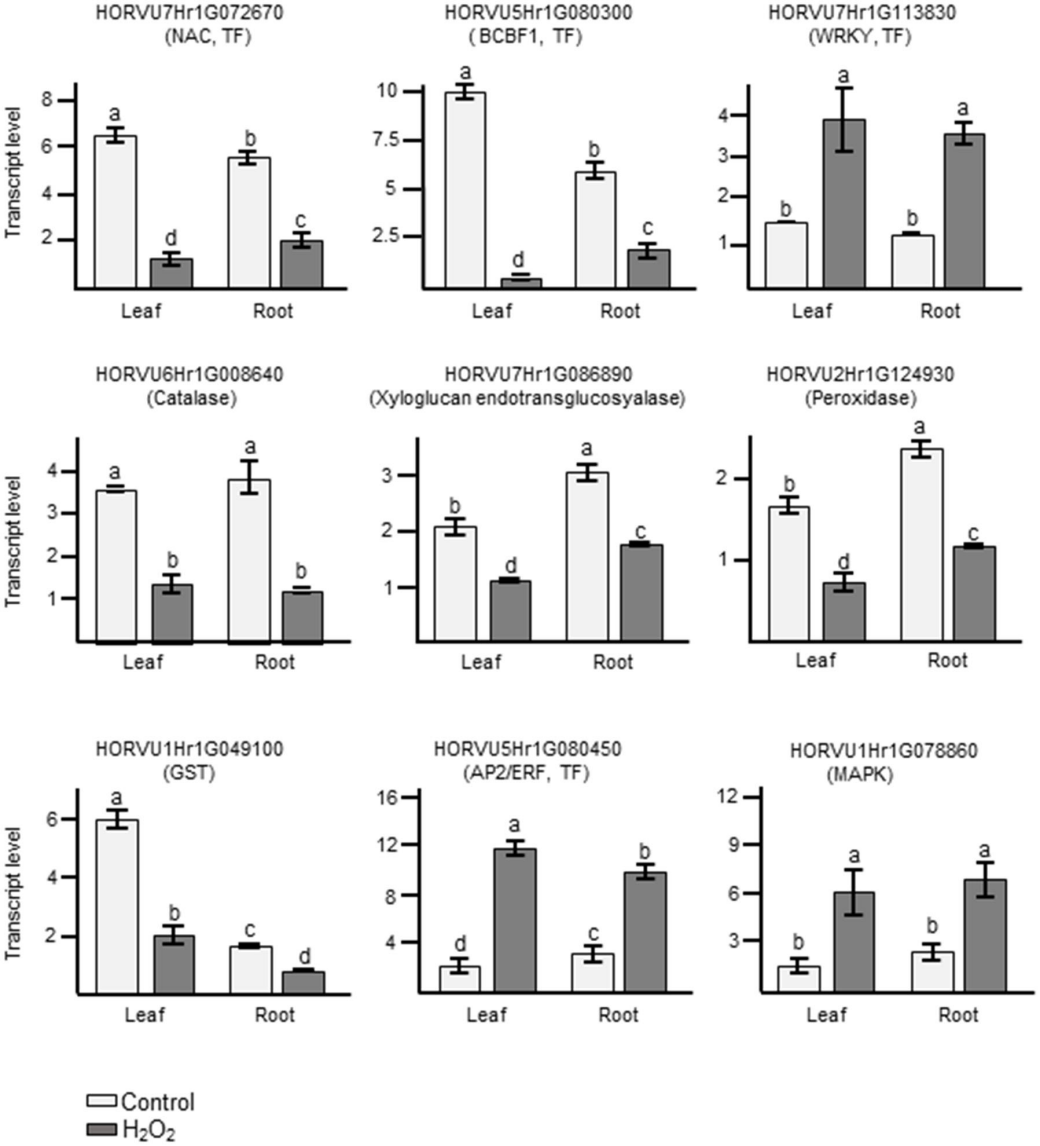
Analyses of transcript levels for selected candidate genes by qRT-PCR. Data represent means ± SE of three biological replicates (n=3), each having two technical repeats. Transcript levels were normalized to *HvACTIN* and *HvGAPDH*. Letters represent significant differences estimated using one-way ANOVA and Tukey’s Post-Hoc HSD test (P<0.05). Potential functions of the genes inferred from orthologous genes in Arabidopsis are indicated in brackets.

In order to confirm the results obtained from RNA-seq analyses, we performed quantitative RT-PCRs (qRT-PCR) on some of the identified DEGs. For these, we selected several DEGs that showed common regulation in leaves and roots in our dataset and which, based on their functional annotation, could be related to oxidative stress (**Supplementary Table S5**). Orthologs to some of them had already been shown to play an important role in H_2_O_2_ and ROS-related signaling not only in Arabidopsis but also in important crops like wheat, maize, and rice (Polidoros et al., 2005; Mylona et al., 2007; Steffens, 2014; Dudziak et al., 2019). They also represent different levels of regulation, some being among the most highly up- or down-regulated genes and other showing a much more subtle response. These genes represent different gene ontologies, and encode for a catalase, a peroxidase, a glutathione S-transferase, several TFs, a MAPKKK, and a xyloglucan endotransglucosyalase, a protein involved in cell wall modification. As shown in **Figure 7** and in **Supplementary Table S5**, the log_2_FC changes observed with the different techniques were often quite close and, in all cases, the results of the qRT-PCR matched the trend observed in the RNA-seq data.

## 4 Discussion

In plants, H_2_O_2_ is a crucial ROS which plays a dual role as a harmful by-product of cell metabolism and as a secondary messenger that affects development and growth. Complex cross-talk between H_2_O_2_ and other signaling molecules, such as Ca^2+^ ions and hormones, plays a key role in regulating different biological processes that contribute to the response to various biotic and abiotic stresses (Peiter, 2016; Saxena et al., 2016). Despite its importance, very little is known about H_2_O_2_-induced changes of the transcriptome in barley. In this study, an analysis of the barley transcriptome in response to H_2_O_2_ was performed using next generation sequencing. First, a suitable concentration of H_2_O_2_ that was shown to initiate a stress response in barley was selected on basis of previously performed experiments (Dodd et al., 2010; Giridhar et al., 2022). An increase in cytosolic Ca^2+^ ([Ca^2+^]_cyt_) is one of the first responses of plants to most biotic and abiotic stresses (Dodd et al., 2010) that in turn leads to downstream stimulus-specific cellular responses. H_2_O_2_ was shown to induce such transient changes of [Ca^2+^]_cyt_ with 10 mM eliciting the highest response in barley roots and leaves (Giridhar et al., 2022). Staining of intact plants with the ROS indicator H_2_-DCFDA confirmed that the exogenously applied H_2_O_2_ penetrated into both organs (**Figures 1B, C**, **Supplementary Figure 1**). To exclude natural degradation of RNA and changes of the transcriptome driven by processes such as senescence or tillering, five-day-old barley plants were used. Growth of monocotyledonous leaves is initiated from the base and the leaf blade shows developmental gradients, i.e., disappearance of poly (A+) RNA levels along the developing blade (Hellmann et al., 1995). Moreover, plant senescence is a natural process known to be initiated by ROS that in turn activates transcription factors interacting with senescence associated genes (Bieker et al., 2012; Shimakawa et al., 2020). Thus, the growth conditions and plant age used in the analysis ensure as much as possible a solely treatment-dependent change of the transcriptome.

Overall, the RNA-seq analysis showed that under the chosen conditions H_2_O_2_ caused more transcriptional changes in roots compared to leaves (**Figure 2**). Most of the identified DEGs were found exclusively in one of the two plant parts, further confirming organ-specific responses. While this difference may be in part due to a difference in H_2_O_2_ penetration into roots and leaves, it is more likely caused by differential response of the two tissues to H_2_O_2_ signals and/or oxidative stress. Only about 10% of the DEGs were found to be up- and down-regulated in leaves as well as in roots, some of which showed counter-regulation. This difference in response is also mirrored by the GO terms associated with the identified DEGs that only showed a minor overlap (**Figure 3**).

### 4.1 Leaf-specific transcriptomic changes in response to H_2_O_2_

Our data showed that several genes encoding for small heat shock proteins (SHSPs) were up-regulated by H_2_O_2_ in barley leaves (**Table 2**). In barley, the roles of several HSPs in response to a diverse range of abiotic stimuli have been characterized (Hlaváčková et al., 2013; Chaudhary et al., 2019; Landi et al., 2019). HSPs have also been shown to play crucial roles during abiotic stresses such as cold and heat in other important crop genera, like rice, maize, and wheat (ul Haq et al., 2019). SHSPs are a subgroup of HSPs defined by their size and a conserved α-crystalline C-terminal domain. They are known to form oligomeric complexes and prevent denatured proteins from aggregation until they can be refolded by other HSPs. They have been speculated to interact with transcription factors of the HEAT SHOCK FACTOR (HSF) family to create the HSP-HSF complex, alteration of which can drive essential reactions in response to ROS (Driedonks et al., 2015). The SHSPs in our data set belong to subfamilies with close orthologs in Arabidopsis, i.e. HSP17.6, 15.4, 15.7, and 17.4 (Li and Liu, 2019). HSP17.6 and HSP15.7 have been shown to be localized in the peroxisomes in Arabidopsis (Ma et al., 2006; Li et al., 2017). Peroxisomes are one of the main subcellular compartments in which ROS are produced by processes such as ß-oxidation and photorespiration, and which are crucial for antioxidant defense (Sandalio et al., 2013; del Río and López-Huertas, 2016). Additionally, *HSP17.4* and *17.6* have been shown to exhibit increased transcript levels during periods of abiotic stress in Arabidopsis (Swindell et al., 2007). Thus, the induction of these HSPs points to a potential role of these proteins in increasing the tolerance to oxidative stress also in barley leaves. The single down-regulated SHSP is an ortholog to *AtHSP15.4*, for which this contrary behavior upon stress was already described (Siddique et al., 2008).

Not surprising, considering the well-established juxtaposition between ROS production and photosynthesis, the application of H_2_O_2_ negatively affected several photosynthetic components (**Table 2**). The most affected group represents chlorophyll a/b binding proteins orthologous to various light-harvesting complex proteins of the LHCb-type and to a component of the light-harvesting complex I, LHCa1, of Arabidopsis. Down-regulation of LHCb-type proteins upon oxidative stress has been previously described (Staneloni et al., 2008). It is likely part of an established photoprotection mechanism to alleviate increased ROS levels generated when the photosynthesis reaction becomes unbalanced, e.g., under high light conditions.

The role of phytohormones like ABA and jasmonate in response to several biotic and abiotic stimuli has been extensively studied in plants (Verma et al., 2016). In our data, several genes related to jasmonate signaling were found to be down-regulated (**Table 2**), including an ortholog of Arabidopsis *12-OXOPHYTODIENOATE REDUCTASE* (*OPR*). The OPR3 protein of Arabidopsis has been denoted as one of the most crucial enzymes in jasmonate synthesis, which converts 12-oxophytodieonic acid (*cis*-OPDA) to OPC8:0 in peroxisomes (Bittner et al., 2022). However, recent studies highlighted the role of an OPR3-independent pathway for jasmonic acid (JA) biosynthesis, involving an OPR2-mediated alternative bypass *via* dinor-OPDA (dnOPDA) and 4,5-didehydro-JA, which is then converted to JA (Chini et al., 2018). Interestingly, we found a down-regulation of the barley ortholog of *OPR2* in leaves, the consequence of which remains speculative due to the unclear role of the OPR3-independent bypass pathway. By contrast, genes coding for *ALLENE OXIDE CYCLASE* (*AOC*) and *ALLENE OXIDE SYNTHASE* (*AOS*) were up-regulated in leaves. These enzymes catalyze the generation of both *cis*-OPDA and dnOPDA, which in turn would increase OPDA production for both pathways. This is interesting, because OPDA is believed to have an independent regulatory function both on transcription (similar to JA-Ile), but also on protein activity by OPDadylation. Moreover, OPDA-mediated signaling seems closely associated with thiol metabolism and redox-mediated processes (Böttcher and Weiler, 2007; Ohkama-Ohtsu et al., 2011; Bittner et al., 2022). Also related to jasmonate signaling are two TIFY domain-containing proteins that were induced in response to H_2_O_2_ (**Table 2**). The TIFY domain is found in members of the JASMONATE ZIM DOMAIN (JAZ)-type transcriptional repressors involved in jasmonate signaling (Chung and Howe, 2009; Pauwels and Goossens, 2011). However, no regulation of TFs associated with jasmonate signaling was detected in our data set.

By contrast, many of the genes associated with other phytohormones, e.g. auxins and ABA, encode TFs or other proteins involved in transcription regulation (**Table 2**). Several of these genes belong to the large family of AP2/ERF-type TFs, members of which have been associated with environmental stresses including hypoxia and oxidative stress. While mostly associated with ethylene, AP2/ERF function is also connected to ABA, gibberellic acid, cytokinin, and brassinosteroids (Xie et al., 2019). The largest group of genes associated with hormones relates to auxin (**Table 2**), the role of which is mostly associated with development and growth. However, experimental evidence linked auxin also to oxidative stress, especially auxin-mediated stress-dependent cell proliferation including the RSL-type TF ROOT HAIR DEFECTIVE SIX-LIKE4 (RSL4) that targets NADPH oxidases also known as respiratory burst oxidase homologs (RBOHs) and secreted plant-specific type III peroxidases that impact apoplastic ROS homeostasis and in turn stimulate root hair cell elongation (Pasternak et al., 2005; Iglesias et al., 2010; Mangano et al., 2017).

### 4.2 Root-specific transcriptomic changes in response to H_2_O_2_

In roots, many DEGs were found to be associated with the detoxification of H_2_O_2_ (**Table 3**), especially peroxidases and genes related to glutathione metabolism. *GLUTATHIONE TRANSFERASES (GSTs)* and *GLUTATHIONE PEROXIDASES (GTPs)* have both been shown to be involved in plant stress responses (Bela et al., 2015; Nianiou-Obeidat et al., 2017). However, somewhat surprisingly, our data showed clear down-regulation of several *GSTs* and *GTPs* along with other key players associated with H_2_O_2_ detoxification such as orthologs of Arabidopsis *ASCORBATE PEROXIDASE* 1 (*APX1*) and *CATALASE 1(CAT1)*. Moreover, two putative *DETOXIFICATION EFFLUX CARRIERS/MULTIDRUG AND TOXIC COMPOUND EXTRUSION* (DXT/MATE) proteins were strongly up-regulated in roots. The MATE family proteins facilitate the efflux of various compounds including substances, such as hormones or flavonoids, that improve adaptation to stress (Ku et al., 2022).

The largest set of genes whose expression was affected in response to H_2_O_2_ belongs to class III plant type peroxidases (**Table 3**), whose role in plant defense mechanisms in response to a wide variety of biotic and abiotic stresses is well established. They play an important role in the cellular redox homeostasis upon stress. In addition, they also catalyze the oxidation of a variety of substrates and have been linked to processes involved in cell wall stability, including lignin and suberin polymerization in response to stress (Kidwai et al., 2020). Thus, the up-regulation of these peroxidases in roots upon H_2_O_2_ treatment is in line with the up-regulation of genes involved in cell wall metabolism observed in this study. Some components of the cell wall architecture, particularly the xyloglucans, have been shown to play an important role in imparting abiotic stress tolerance by coordinating with hormonal and other signaling cascades. For example, a xyloglucan galactosyl transferase from Arabidopsis, SHORT ROOT IN SALT MEDIUM 3 (RSA3), was shown to play a crucial role under salt stress by assembling actin microfilaments and thus preventing ROS accumulation induced by disruption of actin microfilaments (Cho et al., 2006; Li et al., 2013). Also the role of xyloglucan modifying enzymes along with expansins in loosening and expanding the cell wall network upon abiotic stresses has already been described (Tenhaken, 2015).

### 4.3 Commonly and counter-regulated DEGs in responses to H_2_O_2_

Overall, leaves and roots showed very unique transcriptional responses upon H_2_O_2_ treatment. Not only the number of DEGs was much higher in roots compared to leaves, the change in transcription also affected a quite different set of genes (**Figures 2**, **3**). Nevertheless, there are DEGs that were found in both plant parts (**Figure 4**). These 349 DEGs were further divided into four clusters, depending on their expression pattern. Looking at the two larger clusters, the commonly up- or down-regulated DEGs (**Figure 5**, **Supplementary Table S3** and S4), certain patterns in the functional categories can be observed. Both clusters include TFs from different families. This is not unexpected and highlights their versatility in differentially regulating genes as an important part of all stress responses (Javed et al., 2020). However, of the TFs identified in this study, only few have previously been associated with oxidative stress, such as an Arabidopsis ortholog to *HORVU2Hr1G066080* and *HORVU3Hr1G016320*, the *LOB DOMAIN CONTAINING PROTEIN 41 (LBD41)*, that was previously identified in relation with low-oxygen endurance or high-light-induced increase in H_2_O_2_ (Mustroph et al., 2009; Vanderauwera et al., 2011). However, some were found associated with stresses, such as herbivory, that include ROS-mediated signaling or mutations that cause increased levels of ROS (Paudel et al., 2013; Garcia et al., 2016).

Several transporters were found commonly down-regulated (**Supplementary Table S4** and **Figure 5A**). The aquaporin encoded by HORVU4Hr1G085250 is orthologous to the *TONOPLAST INTRINSIC PROTEIN 4;1 (TIP4;1)* of Arabidopsis and rice. Aquaporins not only transport water but also other molecules including H_2_O_2_. *TIP4;1* from barley was shown to be up-regulated by ABA in roots and gibberellic acid in shoots (Ligaba et al., 2011). Moreover, its expression was also up-regulated upon drought (Kurowska et al., 2019). Also sugar transporters of the SWEET-type and PHT1.7 phosphate transporters have been demonstrated to play a role in abiotic stress tolerance and showed variable expression patterns under stress conditions (Cao et al., 2020; Gautam et al., 2022).

We also found common down-regulation of orthologs to *RECEPTOR-LIKE PROTEIN KINASES(RLKs)* from different subfamilies, i.e., WAK, LLR, CRK and RLCK (**Supplementary Table S4** and **Figure 5A**). Experimental evidence suggests that RKLs are a vital part of the growth-defense trade-off, i.e. by facilitating the cross-talk between different phytohormones (Zhu et al., 2023). However, of the specific *RLKs* found commonly down-regulated in barley leaves and roots, only the pepper ortholog of *WAKL20* was described in relation to stress (Zhu et al., 2023). DEGs connected to various facets of primary metabolism were found commonly up-regulated (**Supplementary Table S4** and **Figure 5B**). While several of them are involved in pathways that play a role in stress responses, an obvious connection between these specific DEGs is lacking. Overall, even if no clear connection to oxidative stress exists, many of the commonly regulated DEGs have been described or postulated previously to be involved in stress tolerance mechanisms.

A very small number of DEGs was found counter-regulated upon treatment with H_2_O_2_ (**Supplementary Table S4** and **Figure 6**), the majority of which showing up-regulation in leaves and down-regulation in roots. Several of those genes are connected to aspects of metabolism and hormone signaling, and some orthologous genes of other plant species, such as *SERAT1*, *OSM34*, and *UGT74D1* of tomato, grapevine and Arabidopsis have been previously connected to stress, ABA signaling, or auxin (Tavares et al., 2015; Jin et al., 2021; Park and Kim, 2021; Liu et al., 2022). Remarkably, this cluster also includes a group of nine *HSPs*, and this different expression in leaves and roots raises questions about their specific role in stress response in the different tissues.

## 5 Conclusions

Plant adaptation to changing environmental cues requires acclimation, enabling them to fulfil their lifecycle. This adaptation is based to a large extent on substantial changes on transcriptional level. Our data reveal that H_2_O_2_ modulates the expression of a wide range of genes within the barley genome. The results provide first insights into the significant role of H_2_O_2_ in altering cellular activities in this important crop species. However, in which manner all these genes are coordinated within the cell to provide an appropriate response during stress-induced H_2_O_2_ increase is an important question that needs to be addressed in further research. Many of them have previously been associated to stress responses in barley or more often *via* their orthologs in Arabidopsis or other crops. This reveals a high degree of similarity in the responses of these plants to situations where cellular H_2_O_2_ levels increase either as a toxic by-product of stress or as a dedicated signaling molecule. Other genes identified in this screen have so far not been associated with stress. As important redox molecules participating in plant cell signaling, developmental processes stress responses, as well as causing oxidative damage, uncovering the effect of ROS generally and H_2_O_2_ specifically on gene expression provides good insights into the molecular mechanisms of oxidative stress responses in barley. Such understanding might increase our ability to improve stress resistance in barley and other crops to optimize crop performance and productivity in present and future environmental climate challenges. Particularly, the highest up- or down-regulated genes in our dataset in both tissues were mostly uncharacterized and information on the exact nature of the genes is missing. These data can be used to guide future studies aimed to functionally characterize novel stress-related genes using state-of-the-art experimental designs including generation of mutants and ectopic expression lines. This will enable us to better understand H_2_O_2_ mediated regulation of adaptive processes not only in barley but also in other crops and might thus support targeted breeding of more resilient crops.

## Data availability statement

The datasets presented in this study can be found in online repositories (https://www.ncbi.nlm.nih.gov/sra/PRJNA973626).

## Author contributions

SB contributed to conceptualization, investigation (responsible for most experimental work), formal analysis (responsible for all bioinformatic analysis), validation, visualization, and writing - original draft as well as review & editing. MG contributed to investigation. BM contributed to validation (qRT-PCR) and writing - review & editing. EP contributed to supervision and writing - review and editing. UV contributed to conceptualization, validation, funding acquisition, project administration, supervision, and writing - review & editing. FC contributed to conceptualization, formal analysis, validation, visualization, supervision, and writing - original draft as well as review & editing. All authors contributed to the article and approved the submitted version.
