# Supplementary figures and images for: Global transcriptome profiling reveals root- and leaf-specific responses of barley (Hordeum vulgare L.) to H2O2
Source: Front Plant Sci. 2023 Sep 12;14:1223778. doi: 10.3389/fpls.2023.1223778 (PMC10523330; doi:10.3389/fpls.2023.1223778)

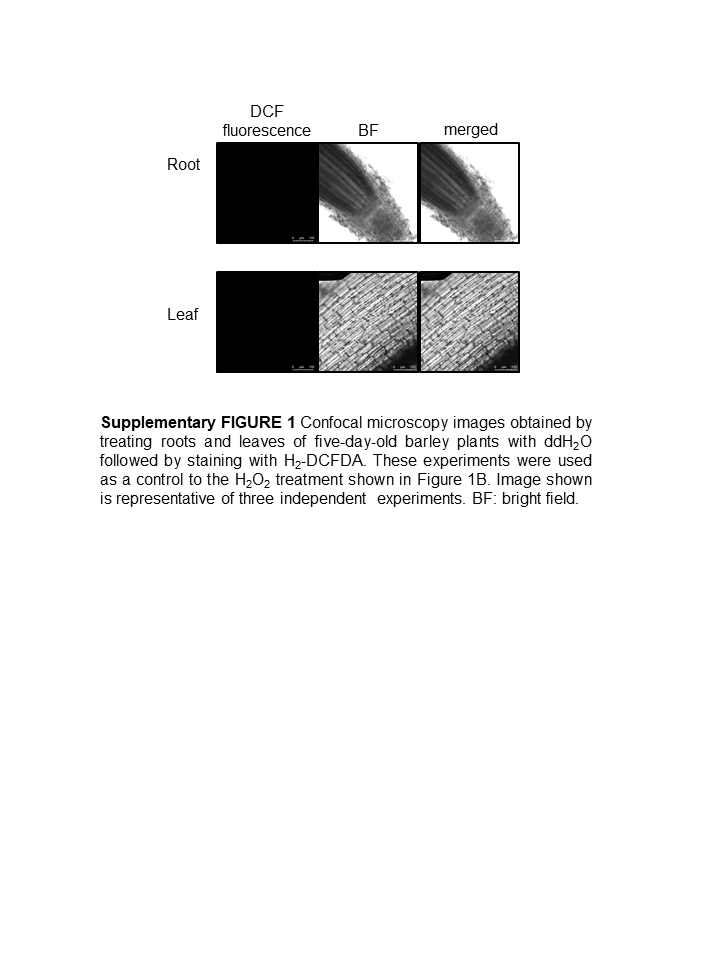

Supplement: Supplementary file 1 [file Image_1.tif]

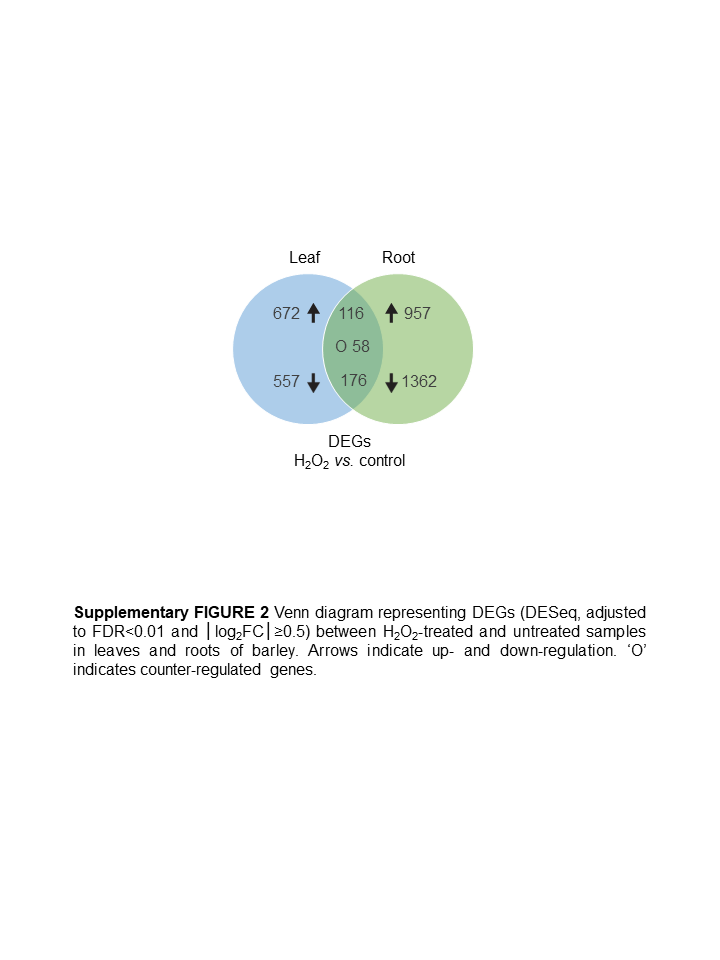

Supplement: Supplementary file 2 [file Image_2.tif]
